# Why Are Christian Schools Popular in Japan Despite the Small Number of Christians? : A Case Study of a Catholic Girls’ Junior and Senior High School

**DOI:** 10.12688/f1000research.168559.2

**Published:** 2026-01-29

**Authors:** Mikiko Yokoyama, Yuuki Matsui, Junko Teruyama, Yoshihiro Goto

**Affiliations:** 1 Institute of Library, Information and Media Science, University of Tsukuba, Tsukuba, Ibaraki Prefecture, Japan; 2 Wako University, Machida, Tokyo, Japan; 3 University of Tsukuba, Tsukuba, Ibaraki, Japan

**Keywords:** Christian schools, cultural capital, school’s founding ideals, qualitative approaches

## Abstract

The proportion of Christians in Japan’s population is very small (0.7%). In contrast, Christian-affiliated schools are numerous and enjoy widespread popularity. As previous studies suggest, Christian schools—especially those for girls—are often associated with positive social images (such as being “refined” or “upper-class”), particularly among young women, and this association has been considered one reason for their popularity. However, much of the existing research is based on statistical analysis, literature review, or quantitative methods, and few studies have employed detailed qualitative approaches.

Some earlier studies have applied Pierre Bourdieu’s theory of class reproduction to analyze the popularity of Christian girls’ schools, but they often frame this in terms of marriage as a pathway to upward social mobility—a perspective that does not fully align with the values of today’s youth, who tend to place greater emphasis on their individual careers. Therefore, this paper focuses on one Christian-affiliated integrated junior and senior high school for girls (referred to as School X) and explores the reasons for its popularity through semi-structured interviews with its alumni. Unlike previous research that has been constrained by gender biases, this study examines the appeal of such schools from the perspective of cultural capital. In particular, it emphasizes the relevance of alignment between the students’ values and the founding mission of the school.

The findings reveal that the students perceived School X’s education as directly contributing to the acquisition of various forms of capital and habitus, as defined by Bourdieu (although not articulated in such terms by the students themselves). Additionally, These results indicate a strong sense of coherence between the school’s founding ideals and the students’ personal values.

In conclusion, this paper offers insight—through the lens of cultural capital and habitus—into why Christian girls’ schools in Japan continue to be highly regarded.

## 1. Introduction

According to the Religious Yearbook (2024 edition) ( Agency for Cultural Affairs, 2024) published by the Agency for Cultural Affairs, the religious affiliation of the Japanese population is as follows: 47.1% identify with Buddhism, 48.4% with Shinto, and only 0.7% with Christianity. Thus, the number of Christians in Japan is relatively small. Nevertheless, Christian-affiliated schools are numerous and continue to attract many students. Among Catholic institutions, there are 148 elementary, junior high, and high schools affiliated with the Federation of Catholic Schools in Japan, and 29 universities and junior colleges under the Catholic University Association ( Japan Federation of Catholic Schools, 2025a, b, c). For Protestant institutions, 102 educational corporations are affiliated with the Federation of Christian Schools in Japan, many of which manage multiple schools ( Association of Christian Schools in Japan, 2025). Regarding the junior and senior high schools addressed in this paper, about 1.3% of all junior high schools and about 4.4% of all senior high schools are Christian-affiliated schools ( Japan Federation of Catholic Schools, 2025a, b; Association of Christian Schools in Japan, 2025; School Basic Survey Annual Statistics, 2025). With the exception of theological seminaries and a few other institutions that train clergy, Christian belief is not a requirement for admission; nor is conversion to Christianity expected during enrollment.

Japan’s education system comprises six years of elementary school, three years each of junior and senior high school, and four years of higher education. Elementary and junior high school education is compulsory, and public schools at these levels are generally tuition-free. While public senior high schools and universities maintain relatively low tuition fees, Christian-affiliated schools are private. Private schools typically require higher fees. Despite this cost, Christian schools remain popular. In the Tokyo metropolitan area—including Tokyo, Saitama, Chiba, and Kanagawa—many students take competitive entrance exams to enroll in Christian-affiliated integrated junior and senior high schools, even though admission to municipal junior high schools, which offer compulsory education, is non-selective.

This paper focuses on an all-girls Christian-affiliated junior and senior high school in Japan. Through interviews with graduates of this institution, the study explores why Christian schools enjoy such popularity despite the low number of Christians in the country. As existing literature suggests, Christian schools are frequently perceived through the lens of an idealized ojō-sama (refined daughter from a good family), particularly among young women, and are often associated with admiration and a positive social images, which are thought to be one of the reasons for their popularity. Meanwhile, in this study, we analyze one case, an all-girls Christian-affiliated junior and senior high school in Japan to investigate how the appeal of Christian education may be linked to the acquisition of cultural capital, based on Bourdieu’s theoretical framework, thereby examining the reasons for the popularity of Christian schools.

## 2. Previous research

Prior research includes An Empirical Study of Religious Education in Contemporary Japan (1998–1999), a study funded by the Grants-in-Aid for Scientific Research, with Nobutaka Inoue as principal investigator. The report ( Inoue et al., 2001) includes ten essays, but many of the discussions focus only on non-religious schools or compare religious and non-religious schools in general. Even when religious schools are examined, Christian schools constitute only a part of the target schools. *Religion and Education* ( Inoue et al., 1997), edited by Nobutaka Inoue, includes a chapter based on a questionnaire survey of students at religiously affiliated universities and high schools. However, this too does not focus on Christian schools.

Yasuko Sato’s work ( Sato, 2006) focuses directly on Christian schools. The book investigates how Christian schools are perceived and how such perceptions relate to the male gaze toward the idealized “destined woman (femme fatale).” Drawing on Pierre Bourdieu’s theory of class reproduction, Sato argues that mission schools that aim to produce “good wives and wise mothers” have historically provided female graduates with the opportunity to attain higher social class through marriage. However, the study mainly analyzes past media content, such as novels, magazines, and comic books, rather than empirically examining people’s actual attitudes.

*Why Are There So Many Beautiful Women in Mission Schools?* ( Inoue et al., 2018), with Shoichi Inoue as the lead author, is another significant prior study. Among its four chapters, Inoue’s “Foreword” and Chapter 1, Protestant Schools Should Not Be Underestimated, take an essayistic approach, while the rest of the book maintains a scholarly tone. These include Chapter 2, “The Success Story of Mission Universities” (by Guo Nan Yan), Chapter 3, “The Changing Christian Image” (by Shinzo Kawamura), and Chapter 4, “Catholicism That Created ‘Ojōsama’ Schools”. However, the book largely focuses on women at Christian universities, relying mostly on statistical and bibliographic data. There has been no detailed research into the image that contemporary individuals have of it.

Hana Krieg’s 2017 article ( Krieg, 2017), The Role of Christian Schools and Their Educational Significance, is also worth noting. This study discusses Christian schools as spaces where specific values are acquired and embodied, using the example of a prestigious Protestant girls’ Christian middle and high school in Kanagawa Prefecture. However, it is essentially based on literature and does not include any actual investigation of public attitudes. Moreover, neither Krieg’s study nor the literature it reviews addresses the relationship between Christian education and cultural capital.

Another relevant work is Popular Image of Students Enrolled in Christian Schools and the Founding Purpose of Christian Schools – A Study Based on a Survey of Youth Living in Tokyo/Kanagawa Prefectures ( Goto et al., 2025). This paper analyzes a web-monitoring survey conducted in 2020 with 700 individuals aged 15–29 residing in Tokyo and Kanagawa. The study offers three reasons why the popularity of Christian schools is not contradictory to the small number of Christians in Japan. First, stakeholders interpret the founding purpose of Christian schools not in a narrow missionary sense, but in a broader sense of fulfilling a God-given mission. Second, many see the significance of Christian schools in pragmatic terms—such as the acquisition of foreign languages and cultures. Third, Christian education is seen as supportive of a flexible idea of “triple-layered faith ^i^.” However, merely 39.4% of the respondents had affiliation with Christian schools. Among these, only 15.4% had actually attended or were currently attending a Christian school. Although supplemental interviews were also conducted, they were not designed as in-depth qualitative studies. As such, little is known about how individuals who actually attended Christian schools understand the founding purposes of these institutions.

## 3. Research objectives and methodology

This study builds upon the prior research by Goto et al. (2025), with a particular focus on one of their key topics: the founding purposes of Christian schools. Our objective is to explore in greater detail how individuals who attended Christian-affiliated schools perceive the foundational intentions behind these institutions. To achieve this, we conducted a qualitative study using semi-structured interviews with graduates of a Catholic all-girls junior and senior high school (hereafter referred to as “School X”), which is widely regarded as one of the competitive schools in Saitama City, Japan. This study was approved by the Research Ethics Committee of the Institute of Library, Information and Media Science, University of Tsukuba (Approval No. 23-140). All participants provided written consent to be part of the research. In addition, all participants were 18 years of age or older and therefore did not fall under the definition of minors as stipulated by the Committee.

Our goal is to assess whether the stated founding purpose of the Christian-affiliated school aligns with the criteria these graduates used when selecting the school. Through this inquiry, we aim to offer insights into why Christian schools continue to be popular in a country with a relatively small Christian population. Inspired by Sato’s earlier work, we also examine the image of Christian schools as perceived by female graduates—particularly in relation to the acquisition of cultural capital and the possibility of attaining higher social status through that capital. In contrast to earlier eras, when Christian education for women was associated with upward mobility via marriage and the cultivation of the “good wife and wise mother” ideal, contemporary young women may view Christian schools as a space where they can independently acquire cultural capital and chart their own paths toward elevated social positions. We pay close attention to this shift in the context of contemporary gender politics.

When students perceive the founding mission of the school to be aligned with their own reasons for choosing it, and believe that this alignment supports the acquisition of cultural capital and upward mobility through personal effort, such coherence may be key. This may help explain the enduring popularity of Christian schools in Japan.

In this study, interviews were conducted with recent graduates of School X who were first-year university students at the time of the survey. Goto et al. (2025), a prior study related to this research, conducted a detailed investigation focusing on young people. However, their survey questions emphasized the upward social mobility through marriage to men of the same or higher social status as the informants themselves, as well as cultivating the traditional ideal of the “good wife, wise mother.” This is because it directly inherited the gender bias seen in prior researches such as Sato (2006) and Inoue (2018). On the other hand, the principal author of this study personally felt that the perspectives on gender held by the younger generation of Japanese differ from those of Sato (2006), Inoue (2018), and even Goto et al. (2025). Therefore, in this study, although some of the co-authors are the same as those in Goto et al. (2025), we aimed to minimize gender bias among researchers by assigning female co-authors as the first and second authors, in order to clarify the actual thoughts of contemporary female students.

Interview participants were recruited through the personal network of the lead author. They shared the commonality of being graduates from the same Catholic integrated junior and senior high school and were first-year university students at the time of the survey, but no restrictions were placed based on family environment, parental beliefs, employment status, or other factors that could potentially influence their views on gender. The interviews were conducted online in February 2024, in the format of a group interview. Five university students (all aged 18 or older), who had graduated from School X within the previous year, participated in a semi-structured group discussion. Notably, one of the five participants was a baptized Catholic who had received infant baptism (According to what they said, of the 160-170 students in each grade, there are only two or three Christians in each grade, and in some years there are none at all.).

We chose to focus on those who graduated from a Christian secondary school rather than those who are currently attending Christian universities because Goto et al. (2025) also found that awareness of school culture tends to decline at the university level in comparison to junior and senior high school levels ^ii^. All interview participants were close friends with one another. The conversation lasted approximately three hours.

It should be noted that the narrow sampling frame introduces limitations. The homogeneity of the group may restrict the diversity of perspectives, and the small sample size increases the risk of selection bias. To mitigate these risks, we strived to recruit participants with different religious backgrounds and enrolled in different universities with diverse majors. Nevertheless, the findings should be interpreted with these limitations in mind.

We analyzed the data using the KJ method, a qualitative technique originally developed by Japanese cultural anthropologist Jiro Kawakita. While sharing the inductive nature of the Grounded Theory (GT) in generating theory from data, the KJ method is distinct in its emphasis on spatial arrangement and holistic synthesis.

The process involved four steps: 1) extracting key phrases (labels) from transcripts; 2) spatially arranging these labels to identify non-linear relationships and grouping them by newly perceived affinity rather than pre-defined categories; 3) interpreting the spatial structure to formulate conceptual themes through abductive reasoning; and 4) synthesizing these concepts into a logical narrative. This approach allows us to move beyond simple categorization and capture the complex, underlying structures of the participants’ experiences ( Scupin,1997; Shimura, 2005).

In Japan, the KJ method is widely known both academically and in business scenes as a brainstorming technique, and it is also commonly used as a method of qualitative research. His books, *Hassōhō: Sōzōsei kaihatsu no tame ni (The method of idea generation: For creativity development) (* Kawakita, 1967/2017) and *Zoku hassōhō: KJ-hō no tenkai to ōyō (Continuation of the method of idea generation: Development and application of the KJ method)* ( Kawakita, 1970), have established an unshakable position as bestsellers and long sellers in Japan.

## 4. Cultural capital, economic capital, social capital, and symbolic capital

In this study, we adopt the concept of cultural capital as theorized by Pierre Bourdieu ( Bourdieu, 1990a, b; Bourdieu, 1992) in order to analyze how graduates of a Christian-affiliated girls’ junior and senior high school perceive the value of their school experience and the social advantages it may confer. Cultural capital refers to the cultural elements that contribute to the reproduction of social inequality in a given society ( Iso, 2022). Alongside cultural capital, Bourdieu identifies economic capital and social capital as additional dimensions of social stratification. The former consists of financial assets, while the latter includes networks, relationships, and social connections.

In addition to these three, Bourdieu introduces the notion of symbolic capital, which encompasses honor, prestige, and authority as socially recognized forms of value. Symbolic capital may overlap with cultural, economic, or social capital, depending on the field (champ (French)) in which it is deployed. For example, academic credentials may be perceived as cultural capital in one context and as symbolic capital in another.

Applying this framework to the context of Christian schooling, we argue that students may internalize the religious knowledge and cultural expectation embedded in such schools and, through this process, acquire resources that can provide them with advantages in their future social and professional lives. In this sense, Christian schools may serve as institutions that not only transmit religious and moral values but also enable the accumulation of cultural capital, foster social capital through alumni networks, and grant symbolic capital via the school’s institutional reputation.

Bourdieu further conceptualizes capital in relation to two interdependent constructs: field (champ) and habitus. A field refers to a social arena in which actors engage in competition over specific forms of capital. Each field operates according to its own logic, values, and stakes, and to succeed in a field, one must understand and conform to its internal rules. Field thus functions as a site of struggle for dominance and legitimacy, and capital can be considered the currency by which actors compete ( Bourdieu,1990a, b; Bourdieu, 1992).

Habitus is defined as a system of durable, transposable dispositions—dispositions being internalized patterns of perception, evaluation, and action. Habitus is produced by the trajectory (trajectoire) of an individual’s life as shaped by their position within and across multiple fields. At the same time, habitus also actively reproduces and reshapes the structure of those fields and the distribution of capital within them. It thus functions as what Bourdieu calls a “structure-generating structure” ( Bourdieu,1990a, b; Bourdieu, 1992).

The relative position of an individual within a given field depends not only on the quantity and type of capital they possess but also on how their habitus allows them to mobilize and deploy these forms of capital. Importantly, Bourdieu’s introduction of habitus enables a non-deterministic understanding of social action, incorporating a degree of fluidity into the otherwise rigid logic of structural positioning. In this view, the strategies actors adopt in the field—their practices, aspirations, and calculations—are shaped by their habitus, which itself is formed through historical participation in particular social environments.

Reconsidering the notion of capital through the interrelations among capital, field, and habitus leads to an important theoretical insight: the same resource may take on different meanings depending on the field. A luxury car, for example, may function as cultural capital in a field where taste and lifestyle are emphasized, while in another it may be read as a straightforward expression of economic capital. In short, whether a given resource operates as cultural, economic, social, or symbolic capital can only be determined through analysis of the triadic relationship among the resource, the field in which it is situated, and the habitus of the actor who uses it. Consequently, Bourdieu argues that there is no such thing as a universally valid medium of capital—economic, social, cultural, or symbolic—that functions in the same way across all fields. Capital is always field-dependent and socially constructed.

In this study, when we refer to “cultural capital, social capital, or symbolic capital acquired through Christian schooling,” we mean those forms of capital that graduates are able to recognize, activate, and utilize in the various fields they enter after graduation. Cultural capital, in this context, includes knowledge, skills, and internalized values; social capital is embodied in relationships and networks such as alumnae associations; and symbolic capital is found in the external recognition and prestige associated with the school’s name and tradition.

Moreover, we argue that the graduates’ ways of engaging in social competition within these fields are shaped by their habitus—dispositions formed and reinforced through the values and experiences of their Christian education. Habitus not only informs their actions but may also serve as a guiding principle that enables them to imagine and enact alternatives to existing social hierarchies. While the pursuit of higher social status may in earlier generations have occurred through marriage, contemporary graduates may instead envision self-determined pathways that involve the strategic deployment of capital accumulated through education.

In what follows, we examine how graduates of School X recognize and activate the various forms of capital they acquired during their schooling, and how these capitals function in the social fields they now inhabit. We also analyze the role of habitus—formed through Christian schooling—as both an embodied legacy of their educational experience and a generative principle for further acquisition of cultural capital throughout the life course.

## 5. Capital acquired through schooling

### 5.1 Cultural capital: Knowledge and cultural acquisition

Our interviews reveal that the participants believe they acquired specific knowledge about Christianity through their attendance at a Christian-affiliated all-girls junior and senior high school. Student A, for example, noted that morning assemblies, which included reading passages from the Bible and reciting the Lord’s Prayer, and classes specifically on biblical texts, were among the distinctive features that set Christian schools apart from secular ones. Student C agreed, suggesting that these experiences provided Christian school graduates with richer “knowledge and ways of thinking about religion.”

In addition, Student D—herself a Catholic who had received infant baptism—highlighted differences in understanding between those who attended Christian schools and those who did not, particularly with regard to religious content embedded in school events. These comments indicate that the respondents identified biblical study, school-wide rituals, and religious ceremonies as core components of Christian education and as primary avenues through which they developed an understanding of Christian values. (As we will discuss in Section 5.3, these educational experiences also played a significant role in the formation of their habitus.)

The participants were also aware of how this knowledge benefitted them beyond secondary education. First, they saw it as an academic asset. For example, Student E, who is studying architecture, found her knowledge of Christianity particularly useful when learning about church architecture. She noted that her understanding of Christian concepts enabled her to design spaces intended for prayer and contemplation. Similarly, Student D, who is majoring in literature, reported that her knowledge of Christian theology helped her interpret Western films in a university class. This kind of interpretive competence extends beyond formal education: as she explained, Christian frameworks aid in understanding foreign cultures and interpreting texts from different cultural backgrounds.

Student A, a member of a university drama group, reported that her knowledge of Christianity enhanced her interpretation of plays, especially those with religious themes. She also mentioned that this knowledge deepened her enjoyment of Christian-themed films and dramas. Student E added that during a visit to Europe, her prior knowledge allowed her to appreciate religious architecture and art more deeply. More broadly, experiences gained through an understanding of foreign cultures—including Christian cultures—can be considered as contributing to higher social status and richer life experiences.

Taken together, these observations suggest that the participants perceived their Christian school education as providing them with valuable cultural resources—particularly religious knowledge and intercultural literacy. While the students may not have consciously labeled this as cultural capital in Bourdieu’s terms, they clearly recognized its usefulness in both academic and broader cultural contexts.

### 5.2 Symbolic capital and social capital: School branding and belonging

The interviews also revealed that the participants felt a strong sense of pride and attachment to their Christian-affiliated all-girls school, and that this pride was partly rooted in the school’s symbolic value and the social connections it fostered. Specifically, the participants identified the school’s brand image—associated with refinement, elegance, and an upper-class sensibility—as a form of symbolic capital, and they recognized the network of shared experience among alumni as a source of social capital.

Student A expressed pride in being a graduate of a “mission school,” referring to the influence of novels and manga in shaping an image of such schools as “somewhat upper-class” and “graceful.” She believed this contributed to the school’s strong brand identity. Student E similarly noted that when she shared stories of unique Catholic school events—such as Christmas celebrations and Bible reading gatherings—people often reacted with surprise and curiosity, which in turn made her feel proud. Student D said that when she mentioned the name of her alma mater, people would respond with, “Oh, that’s an ojō-sama school,” implying a privileged upbringing. She noted that the Christian affiliation added to the school’s classy image. Student B also said she felt pride when asked about her school background, remarking that it was associated with “good upbringing” and “stable, respectable families.”

Student C reflected similar views, stating that her school was considered “an ojō-sama school,” and that people assumed its students were coming from respectable families. She added that her time there had been enjoyable and that she felt proud of her educational background. Taken together, the participants conveyed a shared perception that their school was publicly associated with traits such as “refined,” “graceful,” and “from good families,” and they viewed this association as a form of school branding that carried symbolic capital. Words like “confidence” (Student B) and “fun” (Student C) further suggested a positive emotional attachment to the school and a strong sense of belonging.

This attachment was also expressed through the students’ comments about religious symbols. For example, Student C mentioned feeling “close to Virgin Mary” because there were statues of the Virgin Mary all around campus. Student D added that Mary felt “familiar” and had “blended into daily life.” These comments suggest that familiarity with religious iconography contributed to their emotional and spiritual identification with the school. Shared reverence toward such symbols may have reinforced their sense of belonging to a distinct and cohesive school community.

The interviews therefore demonstrate that the participants were not only aware of their school’s symbolic status but also deeply connected to it through personal experiences and emotional investment. The school’s brand identity—reinforced through both social perception and religious imagery—was internalized as symbolic capital, while the enduring sense of affiliation with the school contributed to the formation of social capital through shared memories, peer networks, and alumni connections.

Accordingly, Christian-affiliated schools like School X provide not only cultural capital, as discussed in Section 5.1, but also symbolic capital through their prestige and reputation, and social capital through the networks and collective identity they foster among students and graduates.

### 5.3 Righteousness, purity, and harmony

The interviews revealed not only that the respondents believed they had acquired knowledge and cultural understanding of Christianity through their school education, but also that they held their own views as former students about what the school intended to instill in its pupils. The stated educational objective of the school was to cultivate the principles of “Sei, Jō, Wa” (Righteousness, Purity, and Harmony)—that is, to foster being one’s true self (Sei), becoming a free individual (Jō), and living together with others (Wa). Acquiring such a way of thinking can be understood as gaining a guiding principle for one’s life, and can itself be regarded as the acquisition of significant cultural capital that may yield benefits later in life. In other words, the students internalized and embodied “Sei, Jō, Wa” as habitus; and this habitus itself functioned as cultural capital, while at the same time, the establishment of such a habitus contributed to the further accumulation of cultural capital through self-directed learning and personal development.

According to the commemorative publication of School X, written by the principals at the time, these educational goals are synonymous with the idea of valuing each individual. That is, the aim is to help students understand the importance of not comparing themselves to others, but rather accepting themselves as they are (Jō), accepting others and living together with them (Wa), and striving to live as their true selves (Sei). At school orientation sessions, the principal would explain this philosophy through a metaphor: “A carrot is a carrot, a radish is a radish, and a burdock is a burdock.” This allegory conveys the importance of not evaluating oneself in comparison with others, but of accepting oneself as one is, accepting others with their unique individuality, and cooperating with others to live out one’s true self.

The following sections will examine how these ideas were manifested in the interviews with the students.

5.3.1 Understanding and embodiment of school values

The interviews first revealed how the respondents understood the concept of Sei, Jō, Wa. Student C stated that the school motto was “Sei as one’s true self, Jō as a free individual, and Wa as mutual support.” Student A interpreted Sei and Jō as “living faithfully according to the role given by God.” Student E explained that Wa meant “togetherness.” These understandings had been shaped by repeated exposure—such as hearing them “frequently from the principal” and “often during assemblies.” Thus, it became evident that the students had acquired knowledge of what Sei, Jō, Wa meant through their school education.

Of course, as reflected in Student A’s statement that she “has not yet reached the point of truly living righteously,” the knowledge of what the educational goals mean is not the same as embodying, habituating, and practicing them. However, the students seemed to believe that they not only understood the meanings, but had also made a habit of being mindful of them, acting in accordance with them, and practicing them in daily life.

Student B said she consciously kept Sei, Jō, Wa in mind during her junior and senior high school years, especially when listening to the principal speak at assemblies. Student C shared that “in terms of Wa, there were many opportunities for mutual help, both in school events and everyday life.” Student E said that she was able to practice Wa, especially in the sense of getting along with everyone, through participating in school events. Student D referred to the vice principal’s slogan “Be your best and truest self” as a guiding principle for practicing Sei, Jō, Wa, and noted that she spent her six years at the school with this consciously in mind.

From these comments, it is clear that the students regarded merely knowing what Sei, Jō, Wa meant as insufficient; they believed it was essential to embody and practice these values in their school life.

Considering that Student D, a Catholic who had received infant baptism, stated, “Personally, I didn’t really know what Christianity was until I entered junior high school,” it can be said that regardless of whether the students were believers or not, they tried to internalize and embody the ideals of Sei, Jō, Wa that the school aimed to instill. The respondents believed that they had acquired these values through educational practices such as school events and assemblies—and particularly in the case of Wa, through the practice of organizing and participating in school events.

In sum, the respondents acquired knowledge of Sei, Jō, Wa through their schooling and, by putting it into practice and internalizing it, incorporated these ideas into their own habitus. As embodied dispositions, Sei, Jō, Wa function not only as habitus but also as cultural capital that facilitates the accumulation of further cultural capital.

5.3.2 Living as One’s True Self

The interviews revealed that the respondents not only understood the idea of “living as one’s true self” as knowledge, but were also consciously aware of it and putting it into practice. They interpreted the “mission” of a mission school as a personal mission to be fulfilled by living as their authentic selves, and were actively searching for that mission.

Student A said: “For example, scissors aren’t for hitting people or writing characters—they’re meant to cut paper. That’s their mission, I was told. So I think the same applies to human beings. Maybe that’s why we were born—that might be our mission. A purpose given by God, perhaps. I think there’s a message behind it: that we should live out and accomplish the mission we’ve been given.” Regarding her own mission, she said, “I’m sure I have one, but I haven’t found it at all yet,” and expressed a desire to try many things in order to discover it.

Student D also said that she felt she was still in the process of searching for her mission, and hoped that by earnestly pursuing what she wanted to do, she might be able to find it. Student B expressed the view that by wandering through life, one would eventually come to see one’s true self. In this way, Student D is “searching while uncertain,” and Student B is “wandering” as she explores her possibilities. These accounts indicate that the respondents are in the process of practicing the idea of “living as one’s true self.”

What is additionally noteworthy is the idea that they feel guided by something toward fulfilling their mission. Student D said that the department she was accepted to during university entrance exams turned out to suit her very well, and she felt that there might be something fateful in that—something connected to her mission. She described this as a “mysterious bond.” This idea of being “guided” toward a mission is evident in Student D’s account.

This notion of “searching for and practicing one’s true mission” was also reflected in their criteria for choosing a university. Apart from the fact that Student D, a Catholic, had once considered enrolling in a Catholic university, and that Student B’s parents had a favorable view of Christian schools and encouraged her to consider a Christian university, Christian affiliation was not a criterion in their university selection. Student E said she chose her university based on her major, and Student C said she decided based on the faculty. These responses suggest that the students took university entrance exams while exploring what they wanted to do, and that their primary criterion was “what they wanted to study.”

The interviews also revealed that one factor supporting such criteria for university selection and encouraging students to live as their true selves was the career guidance provided at X School. Student E said she was more focused on choosing what she wanted to do rather than aiming for a prestigious university, and was happy that her teachers accepted and encouraged that choice. Student D, while expressing some dissatisfaction with the limited academic support for university entrance exams, appreciated X School’s laissez-faire attitude (“you can consult them, but nothing is forced”). She added that, because she was poor at math, if she had been forced to apply to a national university where math is required for entrance exams, she might have suffered mentally and would not have been able to attend the university she is currently at.

Student C said that, in contrast to cram schools that “pressure” students into applying to prestigious universities, she felt reassured by X School’s hands-off policy, which “does not outright deny or force students into any particular path but instead respects their autonomy.” Student A commented that there was no atmosphere of being pushed toward the University of Tokyo (the most prestigious university in Japan) or medical schools, and that she was given the freedom to decide for herself and the time to broadly explore what academic field she wanted to pursue. Student B also expressed a positive opinion about the school’s hands-off approach. From these accounts, it is clear that the respondents viewed X School’s career guidance not as a mechanism to push them toward prestigious or competitive schools, but as a means of expanding their options and offering advice that helped them decide their own paths.

In summary, X School’s education, which broadened their perspectives through advice that increased their options and allowed them to determine their own paths, gave the respondents a sense of living as their true selves and finding their own lives. This awareness was shared by both the Catholic student D and the others who did not share her religious background. In this way, the idea of “living as one’s true self” also contributed to the formation of their habitus and can be considered to function as embodied cultural capital.

5.3.3 Respect for others

The interviews revealed that the respondents believed they were able to exist as their authentic selves at X School precisely because its education did not exclude others who were different and instead embraced diversity. They expressed a desire to see others not through stereotypes, but as individuals with unique personalities.

Regarding the idea of not excluding people who are different, Student C stated that, unlike in elementary school, at X School she was not excluded for having unique opinions, and that she learned this through various experiences, such as the “Wakachiai (share)” event. The ability to express one’s opinion without fear of exclusion also contributed to fostering respect for individuality. Furthermore, as mentioned in 5.3.1, Student E said, “Through cooperating with others during school events, I was able to practice Wa (harmony),” which indicates that she recognized the school’s intentional efforts to ensure no one was excluded during events and activities. When asked in a follow-up group interview, “Which events helped you practice Wa?” Student E responded with examples such as the sports day, chorus contest, and school festival. She noted, “Especially in the school festival, everyone had different roles, and we worked together by utilizing each person’s strengths. I learned to accept others and to accept myself as different from them.”

Next, let us confirm that the respondents felt they could exist as their authentic selves at X School. For example, Student A said, “I was happy that I didn’t have to be confined to the category of being a woman—I could just be a human being.” Student D also realized, after entering university and having some experience in society, that she had not been conscious of her gender during her time at the girls’ school. This suggests that during their junior and senior high school years, they were able to live without concern for gender, and Student D attributed this to the tolerant atmosphere of X School. The notion of freedom within the school culture will be discussed in more detail in the next section.

The interviews also revealed that the respondents did not want to view others through the lens of stereotypes. Regarding gender norms, Student E said, “I don’t want to judge people based on whether they are men or women.” Student D remarked, “It would be ideal to have a way of thinking that is quite neutral, without being overly conscious of gender or things like that.”

This awareness of “not excluding others” may have been shaped by the volunteer activities that were part of X School’s education—such as volunteering at nursing homes, making donations to special education schools, fundraising, participating in river cleanups, and sending Christmas cards.

In sum, it was precisely because they received an education that embraced diversity that the respondents felt they could exist as their true selves and came to want to see others as individuals with their own unique qualities. This indicates that the school education played a role in helping them acquire a mindset of respecting others as individuals. Furthermore, this way of thinking can be understood as contributing to the formation of their habitus and functioning as embodied cultural capital.

5.3.4 A guiding principle for life: Understanding one’s mission

As discussed thus far, the education at X School led the students to believe that it is important not to define themselves by comparing themselves to others, but instead to accept themselves as they are, respect others, and strive to become their true selves. This was understood as the mission to fulfill what one is meant to do as one’s authentic self.

Through various school events, career guidance, talks at assemblies, and daily school life, they came to embody the habitus represented by Sei, Jō, Wa, put that spirit into concrete practice, searched for their true mission, and learned to respect others with individuality. This, in turn, led them to respect themselves as individuals. Acquiring such a mindset means obtaining a compass for living one’s own life.

In essence, the habitus of Sei, Jō, Wa, along with the knowledge and experiences gained through it, becomes effective cultural capital for living one’s life meaningfully—regardless of the social field one may enter after graduation. The foundation for an education that fosters awareness of one’s true mission lies in the “atmosphere of freedom” described in the previous section. In the following section, we will discuss this consciousness and the elements that support it.

## 6. Attitudes toward freedom in school

The interviews revealed that the respondents understood the climate of X School as one that embraces freedom. The term “freedom (within the school’s climate)” appeared in the comments of Students B, D, C, and E. For instance, Student C described freedom as “being in a space where you can live without reservation.” Although Student E did not explicitly define what she meant by freedom, she referred to it in various ways, saying things like “I felt like they valued me as I was” and “a kind of freedom where things aren’t overly enforced.” In recounting her experience with career guidance, Student D also implied this meaning, stating that “it was very free in the sense that nothing was forced.” In this way, the respondents perceived “freedom (within the school’s climate)” as the ability to live freely without coercion.

They also used the word “freedom” in relation to the perceived flexibility of Catholicism in Japan. Student A, whose twin sister attended a Protestant school, said that the religious education at X School seemed “rather lax” in comparison to that at Protestant schools. Student D, a Catholic, referred to her mother’s view that while Protestantism is “strict” and “rigid,” Catholicism is “relatively free,” and added that compared to the Protestant school her brother had attended, “X School was much freer,” indicating that she shared the same view. Furthermore, D’s account of celebrating traditional milestones such as Shichi-Go-San and Seijin-shiki (Coming-of-Age Day) at church, while also visiting Shinto shrines and Buddhist temples with her family, supports the idea that Catholicism is considered flexible and free in this context. These observations suggest that Student D’s emphasis on “freedom (within the school’s climate)” stems from her perception that X School’s culture reflects the flexibility inherent in her own Catholic denomination.

The “freedom” they attribute to the school’s culture fundamentally refers to freedom from coercion. However, both Student A and D whose sibling attended a Protestant school expressed that Catholicism is not only non-coercive but also flexible, and they identified this flexibility as a form of freedom as well. —They viewed the spirit of X School as situated along this continuum of Catholic flexibility.

## 7. The founding purpose of the school

X School was established by a religious order based in Canada. The founding purpose of the school was to educate children so that they could discover and live according to the way of life God desires—that is, to find and fulfill their own mission. The education at X School can be understood as carrying the mission of fostering students’ awareness of the importance of living their own mission and of understanding Christian values. While this may potentially lead to evangelization, the school’s official website states clearly that, although students are educated based on a Christian view of humanity, they are not required to convert. The school’s founding purpose and mission, then, can be said to involve promoting Christian values and behaviors that become internalized in students as part of their everyday lives.

## 8. Reasons why students and parents chose X school for junior high school

What reasons did the students and their parents have for choosing X School at the time of the junior high school entrance exams? While practical reasons such as academic prestige, exam location, and timing were mentioned in the interviews, this section focuses on internal reasons, so those practical factors are not discussed in detail here.

Student D and her parents are Catholics, and she said they encouraged her to attend a Catholic school if she were to go to a Christian school. Other reasons mentioned include favorable impressions from acquaintances who attended the school and positive experiences at school information sessions or cultural festivals. Student C noted that a friend of her brother had been accepted to the school, that it was a girls’ school, and that she knew many students with outgoing personalities. Student E mentioned that she had relatives who attended the school and that she had a good impression of the atmosphere when she visited. Student D said that meeting the principal at a “Christian School Fair” and finding them to be a kind person, as well as the atmosphere at the cultural festival, influenced her decision. Student B decided to take the entrance exam after hearing a senior student’s story at a cram school event the previous year.

From these responses, we can see that the students held a positive image of the school. They referred to acquaintances such as “my brother’s friend,” “relatives,” and “a senior student who passed the exam last year,” as well as the personality of the principal—“I thought the principal was a really nice person” (Student D, also echoed by Students E and C). Additionally, both Students C and A cited the fact that it was a girls’ school as a reason for their choice.

It was also clear that the girls got a feel for the school’s atmosphere by listening to the principal’s story of “Carrot, Radish, and Burdock” at the information session. Student A said that, while other schools emphasize how many students go on to prestigious universities like the University of Tokyo or to medical schools, X School shared stories like that instead, which made her feel the school had a laid-back atmosphere. Student E also felt that this story reflected the school’s acceptance of individuality and described it as “a lovely idea.” Student C commented that, unlike typical school sessions that focused on academic results and university admissions, the speaker from X School did not talk about such matters and that “the principal’s relaxed speaking style was very engaging.” Similarly, Student D noted that while other schools emphasized entrance into prestigious universities, X School uniquely emphasized the value of carrots and such, and her mother—who heard the same story in the waiting room during the entrance exam—said, “I thought it was a very interesting school.” Student B, although not present at the information session, said that after entering the school and hearing the principal’s “Carrot” story, she felt “influenced by the school’s atmosphere.”

Comments such as “laid-back” (Student A), “a nice idea” (Student E), “a relaxed and interesting principal” (Student C), “what a unique school” (Student D), and “influenced by the atmosphere” (Student B) suggest that these students resonated more with the idea of “living as one’s true self” than with discussions focused solely on academic performance or prestige. Additionally, D’s mother’s remark that “it seemed like a very interesting school” suggests that the school’s emphasis on living one’s true self also resonated with parents.

## 9. The founding mission of the school and the intentions of students and parents

As discussed in the previous chapter, X School clearly presents its educational philosophy during school information sessions and other events: rather than measuring oneself by comparison to others, students are encouraged to accept themselves as they are, respect others, and strive to become their true selves. This philosophy is understood not only by students but also by their parents, who consciously choose to enroll their children in the school with these values in mind.

The educational goal of X School is not to produce students who gain admission to prestigious and highly ranked universities, but rather to guide students toward becoming individuals who accept themselves without comparison to others and who respect those around them as they work toward discovering and becoming their authentic selves. Students who understand this goal perceive the school as a relaxed, liberating, and pleasant space, and they are drawn to it out of a sense of shared values. As expressed by Student D’s mother, who described it as “a very interesting school,” parents too resonate with the school’s educational philosophy as conveyed in information sessions, and choose to send their children to X School based on that alignment.

Although Student D was the only one to explicitly comment from a parental perspective during the interviews, it is important to note that, in Japan, it is extremely rare for elementary school students to attend school information sessions alone; they typically go with their parents. Parents who attend such sessions with their children and hear the school emphasize living as one’s true self over university entrance results are more likely to resonate with that message and choose X School over other schools with similar academic reputations or entrance success rates. Considering these circumstances, it is highly likely that the parents who choose X School for their children are seeking an education that fosters the ability to accept oneself without comparison, to respect others, and to aim to become one’s authentic self.

From the above, we can conclude—at least based on this study—that the founding mission of X School aligns with the intentions and values held by both students and their parents.

## 10. Discussion

As our analysis has shown, the interviewees were clearly aware that the knowledge, culture, and Christian values they acquired through their schooling—internalized through practice and embodied as habitus—constitute resources that could prove useful in their future lives. In other words, they recognized that they had gained cultural capital. Yet, from an analytical perspective, it seems likely that the education they received at X School will serve them in even more significant ways than they themselves currently realize.

As discussed in Chapter 5, while the concept of “cultural capital” was not explicitly invoked, the graduates nonetheless interpreted their experiences in terms that align closely with the notion. They viewed the acquisition of knowledge and cultural understanding related to Christianity as an asset, particularly in navigating intercultural contexts. Furthermore, a strong sense of belonging to X School was repeatedly expressed, a form of identification that fosters tightly-knit alumni relations—potentially a source of social capital that may benefit them in building future networks. Additionally, because the school is perceived as having symbolic value in wider society, the experience of attending X School may also function as symbolic capital.

Beyond this, students internalized the school’s core values of Sei (righteousness), Jou (purity), and Wa (harmony)—principles that encouraged them to accept themselves without comparison to others, to value authenticity, and to treat others with respect. This moral and relational orientation constitutes a form of habitus that is not only transferable across social contexts, but also productive: it enables the further accumulation of cultural capital in the form of practical life guidance. In this sense, what they have gained through school—knowledge, dispositions, and a set of guiding principles—may support their future career trajectories and social mobility. Particularly, possessing a clear sense of purpose or “life compass” is crucial for sustainable career formation, including during moments of transition.

As Chapter 7 outlined, the foundational mission of X School is to offer students such a compass—encouraging the development of the Sei-Jou-Wa spirit. While students may choose the school for a range of reasons, including pragmatic ones, our interview data (as explored in Chapters 8 and 9) suggest that alignment with the school’s values played a central role in the decision-making process for many of our participants and their families.

Taken together, these findings suggest that X School’s educational model contributes meaningfully to students’ accumulation of cultural capital. This capital is not only instrumental for personal development and social positioning but also serves as a reason why families choose Christian schools like X School. Moreover, the perception of such schools as providers of valuable cultural capital contributes to the production of both social and symbolic capital, reinforcing the school’s reputation. In this way, our analysis of X School graduates points to a strong alignment between the school’s founding ideals and the expectations and values of its students and their families—a convergence that likely helps explain the enduring popularity of X School in a largely non-Christian society like Japan.

## 11. Conclusion

In this paper, we conducted semi-structured interviews with graduates of a Catholic all-girls junior and senior high school (referred to as “X School”) who were first-year university students at the time of the study. Through this, we examined the relationship between the school’s founding purpose and the criteria by which it is selected as an educational institution. We aimed to clarify, from the perspective of cultural capital, one reason why Christian all-girls schools continue to attract students in Japan, where the Christian population is small.

As a result, we found that in the case of X School, one reason why such Christian schools are highly regarded—despite the small number of Christians in Japan—can be understood through the lens of various forms of capital and habitus, with a particular focus on cultural capital. However, it should be noted that the findings presented here are based on interviews with a specific group of students, and further research is needed to determine whether the results can be generalized to Catholic all-girls schools or Christian mission schools more broadly ^iii^. Furthermore, in order to expand this to Protestant middle and high schools, it will be necessary to consider the similarities and differences between Catholic mission and Protestant calling, as well as the differences in the flexibility of each toward other religions (Morimoto points out the lack of tolerance within the various denominations of Protestantism ( Morimoto, 2018)). From there, we must also consider what Christian middle and high schools have in common.

Moreover, while we inferred and gathered impressions of parents’ perspectives through the graduates’ interview responses, we did not directly investigate the views of the parents themselves. This is another issue that needs to be addressed in future studies.

## Data Availability

The interview data that support the findings of this study contain personally identifiable and sensitive information, including participants’ religious beliefs and practices, and therefore cannot be shared publicly. In the informed consent form, which was approved by the Research Ethics Committee of the Institute of Library, Information and Media Science, University of Tsukuba (Approval No. 23-140), participants agreed that their data would only be accessed by the investigators of this study. Accordingly, the raw data cannot be disclosed to third parties. No further data are available.

## Notes

^i^
The key concept that runs throughout “Why Are There So Many Beautiful Women in Mission Schools?” is the assertion that most Japanese are essentially Christians, Buddhists, and Shintoists without the baptismal process.

^ii^
Moreover, Goto et al. (2025) reported that students who attended Christian schools in the same school system as their elementary, junior high, and high school found that elementary schools had a higher proportion of Christian teachers than junior high and high schools, and that there was a stronger emphasis on emotional education through Christianity.

^iii^
One essay by a graduate of a Catholic middle and high school argues that there was a lack of freedom at the school ( Morimoto, 2021).
