# Supplementary material for: Why Are Christian Schools Popular in Japan Despite the Small Number of Christians? : A Case Study of a Catholic Girls’ Junior and Senior High School
Source: F1000Res. 2026 Feb 19;14:892. Originally published 2025 Sep 9. [Version 3] doi: 10.12688/f1000research.168559.3 (PMC12891952; doi:10.12688/f1000research.168559.3)
Supplement: Peer review history

# Reviewer report: Reviewer response for version 3

**DOI:** 10.5256/f1000research.196592.r460251

**Published:** 2026-04-03

**Authors:** Rudy Pramono

**Affiliations:** 1 Universitas Pelita Harapan, Harapan, Harapan, Indonesia

I have reviewed the revised manuscript (version 3). The authors have substantially improved methodological transparency by expanding the KJ method description and clarifying sampling rationale. The theoretical application of Bourdieu’s capital forms is well-executed. However, the generalizability remains limited due to the small, homogeneous sample (n=5) from a single Catholic girls’ school. A more explicit contrast with prior studies on marriage-based mobility would sharpen the argument for a contemporary value shift. The conclusions are appropriately cautious. Overall, this is a valuable qualitative contribution that is now suitable for indexing after minor tightening of the discussion.

Is the work clearly and accurately presented and does it cite the current literature?

Yes

If applicable, is the statistical analysis and its interpretation appropriate?

Not applicable

Are all the source data underlying the results available to ensure full reproducibility?

No source data required

Is the study design appropriate and is the work technically sound?

Yes

Are the conclusions drawn adequately supported by the results?

Partly

Are sufficient details of methods and analysis provided to allow replication by others?

No

Reviewer Expertise:

Education and tourism

I confirm that I have read this submission and believe that I have an appropriate level of expertise to confirm that it is of an acceptable scientific standard.

# Reviewer report: Reviewer response for version 2

**DOI:** 10.5256/f1000research.195618.r453670

**Published:** 2026-02-10

**Authors:** Rudy Pramono

**Affiliations:** 1 Universitas Pelita Harapan, Harapan, Harapan, Indonesia

This revised manuscript has addressed key methodological concerns from the reviewers, particularly by expanding the description of the KJ method and providing a more transparent rationale for the small, purposive sample. The theoretical framework using Bourdieu’s capital concepts is clearly applied.

 However, the paper would benefit from a more explicit connection between the empirical findings and the broader research question. While the analysis effectively shows how graduates perceive acquired capital, the discussion could more directly link this to explaining the schools' popularity despite low Christian affiliation. A sharper contrast with prior studies focusing on marriage mobility would strengthen the argument about a contemporary shift in values.

 Additionally, the conclusion’s limitations section is appropriate, but a brief reflection on how the specific Catholic, all-girls context might shape the findings—especially the emphasis on “freedom” and flexibility compared to Protestant schools—would add nuance.

 Overall, the study makes a valuable qualitative contribution, and with minor refinements to tighten the argument, it is suitable for indexing.

Is the work clearly and accurately presented and does it cite the current literature?

Yes

If applicable, is the statistical analysis and its interpretation appropriate?

Not applicable

Are all the source data underlying the results available to ensure full reproducibility?

No source data required

Is the study design appropriate and is the work technically sound?

Yes

Are the conclusions drawn adequately supported by the results?

Partly

Are sufficient details of methods and analysis provided to allow replication by others?

No

Reviewer Expertise:

Education and tourism

I confirm that I have read this submission and believe that I have an appropriate level of expertise to confirm that it is of an acceptable scientific standard.

# Response

**Published:** 2026-02-13

**Authors:** Mikiko Yokoyama

**Affiliations:** University of Tsukuba, Tsukuba, Ibaraki Prefecture, Japan

Thank you for your comment.

 On “a brief reflection on how the specific Catholic, all-girls context might shape the findings”

 I have supplemented "Conclusion."

 The relevant parts are the following two points.

 “However, it should be noted that the findings revealed in this study were observed in the case of a particular Catholic girls' school. If we were to put it another way, it should be noted that the findings presented here are based on interviews with a specific group of students, and further research is needed to determine whether the results can be generalized to Catholic all-girls schools or Christian mission schools more broadly.”

 “At that time, it will be necessary to examine how “freedom” is understood in Protestant junior and senior high schools.”

 We also consider comparisons with Protestant junior and high schools to be an important issue and a topic for future study; we are currently drafting a separate paper on it.

 Thank you for your advice.

# Reviewer report: Reviewer response for version 1

**DOI:** 10.5256/f1000research.185746.r416663

**Published:** 2025-11-20

**Authors:** Matthew H Lee

**Affiliations:** 1 Kennesaw State University, Kennesaw, Georgia, USA

I believe I was asked to review this article because of some of my other research on Christian schools (see, for example, Lee, Johnson, & Cheng, 2024, published in the Journal for the Scientific Study of Religion).

 The authors conduct a qualitative study of five recent graduates of a Christian-affiliated school in Japan to understand their reasons for choosing a Christian-affiliated school. While prior research has continued the cultural perceptions of Christian-affiliated schools in Japan, no prior study has used qualitative methods to exclusively study graduates of a Christian-affiliated school to answer this question. This study thus offers many unique and important insights for why an education provided by a Christian-affiliated school can offer public benefit, even for students who do not identify as Christians, and specifically in the Japanese context.

 I have a few minor comments for this otherwise excellent manuscript.

 First, in the introduction, you provide some statistics on the proportion of people in Japan who identify as Buddhist, Shinto, or Christian. You also provide some statistics on the number of Christian-affiliated schools, including Catholic schools and Protestant schools. While the former is presented as percentages and the latter is presented as raw counts, it would be helpful to convert those figures to be able to compare. For example, if 0.7% of people in Japan are Christian, how many K-12 students would we expect to be Christian, or to come from Christian households? If there are 148 primary/secondary Catholic schools, 29 Catholic universities, and 102 Protestant schools, what proportion is that relative to all schools in Japan? How many students do they enroll, and what is that as a proportion of all students in Japan. A more thorough explanation of these statistics would help establish that Christians are few, but Christian-affiliated schools are popular.

 Second, forgive me for saying this if I am mistaken, but you note that the manuscript was translated from Japanese to English using AI. There are a couple word choices that I do not believe you intended. For example, in English, the term "femme fatale" communicates a seductive character that I do not believe is intended in your context. Similarly, the idea that people could be "well-bred" is probably not intended. Perhaps you meant "well read" or that these students came from respectable families?

 Third, I believe the methodology section could be strengthened. In particular, I think more could be said about how these five students in particular were identified. Did the researchers solicit a broader response and only found five subjects that satisfied their inclusion criteria? Did the researchers work in collaboration with the school itself, who recommended these five individuals? Do the researchers merely have convenient personal connections with these five graduates? Please clarify how the sample was chosen.

 Finally, I would recommend reorganizing a few of your sections. In my mind, it would make sense to present the mission of the school as well as the a priori reasons for choosing a school (described in sections 7, 8, and 9) before presenting the post hoc reflections on what benefits these students gained from attending the school (sections 5 and 6). If possible, more explicit language about the founding purpose of the school would be helpful, though perhaps that was omitted for deidentification purposes.

 Again, I learned a lot from reading this work, which I found both interesting and insightful. Congratulations to the researchers!

Is the work clearly and accurately presented and does it cite the current literature?

Yes

If applicable, is the statistical analysis and its interpretation appropriate?

Yes

Are all the source data underlying the results available to ensure full reproducibility?

No

Is the study design appropriate and is the work technically sound?

Yes

Are the conclusions drawn adequately supported by the results?

Yes

Are sufficient details of methods and analysis provided to allow replication by others?

Partly

Reviewer Expertise:

I study the economics of education, in particular education choice policy and religious schools.

I confirm that I have read this submission and believe that I have an appropriate level of expertise to confirm that it is of an acceptable scientific standard.

## References

[1] : How Do Parents Choose Schools for Their Children? Experimental Evidence from the Private Christian School Sector. *Journal for the Scientific Study of Religion* .2024;63(3) : 10.1111/jssr.12911 579-595 10.1111/jssr.12911

# Response

**Published:** 2026-01-22

**Authors:** Mikiko Yokoyama

**Affiliations:** University of Tsukuba, Tsukuba, Ibaraki Prefecture, Japan

We appreciate your valuable feedback.

 Regarding the first point your comments

 It is understandable that the former and the latter were shown in percentages and numerical values respectively, making them hard to compare. Regarding the statistics on the number of schools, the percentages were indicated to make comparisons easier. The following sentences have been added.

 “Regarding the junior and senior high schools addressed in this paper, about 1.3% of all junior high schools and about 4.4% of all senior high schools are Christian-affiliated schools. （Japan Federation of Catholic Schools, 2025a, b; Association of Christian Schools in Japan, 2025; School Basic Survey Annual Statistics, 2025）”

 Regarding the second point of your comments

 We used AI to assist in our initial translation but have checked the output and believe the English word choices to be reflecting the original wording. As for the 2 points that were pointed out - the first one, "femme fatale" was actually not a translation but the exact words used by the author cited (Sato inserted this French term verbatim in the Japanese text) and therefore we choose not to modify this. The second one, "well-bred," is indeed used to refer to the fact that they were coming from a respectable family, but we realize that this is a rather old-fashioned expression in English so we have changed it according to the reviewer's advice. The changes are as follows.

 “Student C reflected similar views, stating that her school was considered “an ojō-sama school,” and that people assumed its students were well-bred.”

 ↓

 “Student C reflected similar views, stating that her school was considered “an ojō-sama school,” and that people assumed its students were coming from respectable families.”

 Regarding the third point of your comments

 We understood that more detailed information is needed regarding the sample selection method. To address this point, we have rewritten part of the fourth paragraph in Chapter 3 as follows. (The following response overlaps with another reviewer's comments (regarding Strengthen Sampling Rationale and Refine Limitations Section)).

 “To understand current perceptions, and in light of Goto et al.’s (2025) focus on youth, we conducted interviews with recent graduates of School X who were in their first year of university at the time of the study.”

 ↓

 “In this study, interviews were conducted with recent graduates of School X who were first-year university students at the time of the survey. Goto et al. (2025), a prior study related to this research, conducted a detailed investigation focusing on young people. However, their survey questions emphasized the upward social mobility through marriage to men of the same or higher social status as the informants themselves, as well as cultivating the traditional ideal of the "good wife, wise mother." This is because it directly inherited the gender bias seen in prior researches such as Sato (2006) and Inoue (2018). On the other hand, the principal author of this study personally felt that the perspectives on gender held by the younger generation of Japanese differ from those of Sato(2006), Inoue(2018), and even Goto et al. (2025). Therefore, in this study, although some of the co-authors are the same as those in Goto et al. (2025), we aimed to minimize gender bias among researchers by assigning female co-authors as the first and second authors, in order to clarify the actual thoughts of contemporary female students. 

 Interview participants were recruited through the personal network of the lead author. They shared the commonality of being graduates from the same Catholic integrated junior and senior high school and were first-year university students at the time of the survey, but no restrictions were placed based on family environment, parental beliefs, employment status, or other factors that could potentially influence their views on gender.”

 Regarding the fourth point of your comments

 Thank you for your suggestion on section reorganization. We understand the point you raised, but since it would require a major revision, we would appreciate it if we could proceed as is this time. Regarding the clear expression of the school's founding purpose, as you pointed out, it has been omitted for anonymization purposes.

# Reviewer report: Reviewer response for version 1

**DOI:** 10.5256/f1000research.185746.r416666

**Published:** 2025-09-29

**Authors:** Rudy Pramono

**Affiliations:** 1 Universitas Pelita Harapan, Harapan, Harapan, Indonesia

To enhance the article's scientific soundness and rigor, the authors should prioritize the following before or during the peer review process:

1. Enhance Methodological Transparency: This is the most critical area for improvement. The authors must provide a detailed description of the KJ method application and make the core interview protocol available.
2. Strengthen Sampling Rationale: Explicitly discuss the limitations of the small, homogeneous sample and describe how the research design attempted to mitigate the associated risks of bias.
3. Refine Limitations Section: Integrate a more thorough discussion of limitations (sample, generalizability, potential for social desirability bias) earlier in the paper, not just in the conclusion.

By addressing these points, the authors will significantly strengthen the manuscript's rigor, transparency, and contribution to the field, making it a stronger candidate for indexing.

Is the work clearly and accurately presented and does it cite the current literature?

Yes

If applicable, is the statistical analysis and its interpretation appropriate?

Not applicable

Are all the source data underlying the results available to ensure full reproducibility?

No source data required

Is the study design appropriate and is the work technically sound?

Yes

Are the conclusions drawn adequately supported by the results?

Partly

Are sufficient details of methods and analysis provided to allow replication by others?

No

Reviewer Expertise:

Education and tourism

I confirm that I have read this submission and believe that I have an appropriate level of expertise to confirm that it is of an acceptable scientific standard, however I have significant reservations, as outlined above.

# Response

**Published:** 2026-01-22

**Authors:** Mikiko Yokoyama

**Affiliations:** University of Tsukuba, Tsukuba, Ibaraki Prefecture, Japan

Thank you for your valuable comments.  We have responded to the points 1 through 3 as follows.

 Regarding Enhance Methodological Transparency(1)

 We agree that a detailed explanation of the KJ method is essential for international readers.

 As per your advice, we have significantly expanded the description in the Methodology section. Specifically, we have clarified how the KJ method utilizes spatial configuration and abductive reasoning to derive themes. We highlighted that, unlike standard coding methods, this approach enables the reconstruction of context and the discovery of novel hypotheses from qualitative data. We believe this addition justifies our choice of method and enhances the transparency of our analysis. The changes are as follows.

 “We analyzed the data using the KJ method, which is well-suited for uncovering themes in qualitative research.”

 ↓

 "We analyzed the data using the KJ method, a qualitative technique originally developed by Japanese cultural anthropologist Jiro Kawakita. While sharing the inductive nature of the Grounded Theory (GT) in generating theory from data, the KJ method is distinct in its emphasis on spatial arrangement and holistic synthesis.

 The process involved four steps: 1) extracting key phrases (labels) from transcripts; 2) spatially arranging these labels to identify non-linear relationships and grouping them by newly perceived affinity rather than pre-defined categories; 3) interpreting the spatial structure to formulate conceptual themes through abductive reasoning; and 4) synthesizing these concepts into a logical narrative. This approach allows us to move beyond simple categorization and capture the complex, underlying structures of the participants' experiences. （Scupin,1997; Shimura, 2005）

 In Japan, the KJ method is widely known both academically and in business scenes as a brainstorming technique, and it is also commonly used as a method of qualitative research. His books, Hassōhō: Sōzōsei kaihatsu no tame ni (The method of idea generation: For creativity development )（Kawakita, 1967/2017）and Zoku hassōhō: KJ-hō no tenkai to ōyō (Continuation of the method of idea generation: Development and application of the KJ method)（Kawakita, 1970）, have established an unshakable position as bestsellers and long sellers in Japan.”

 We will introduce the section related to the KJ method from the abstract of Scupin's paper (1997) titled KJ Method: A Technique for Analyzing Data Obtained from Japanese Ethnology. 

 The KJ method was developed as a result of having difficulties in interpreting ethnographic data in Nepal. The KJ method builds upon Charles Pierce's notions of abduction and relies upon intuitive non-logical thinking processes. Kawakita's methods were developed and diffused throughout Japanese management and educational circles. Kawakita believes that his methodology has the potential of liberating humanity from the mechanistic philosophy imposed by Western Civilization. The KJ method, according to Kawakita has universal applicability and does not only conform to Japanese culture and management decision-making processes which are generally based on the group-orientation model. This simplistic group-orientation model of Japanese society is being criticized by anthropologists such as Harumi Befu. Kawakita argues that human nature is universal, and that the KJ method, as a means of decision-making can be utilized in all societies throughout the world to implement social and economic development.

 Furthermore, Shimura (2005) explains the KJ Method as follows.

 In this paper, the KJ method is positioned as a method of performing abductive reasoning from observations of the real world in "Field Science."

 1)The Essence of the KJ Method: The Method for "Abduction"

 ・Jiro Kawakita, the developer of the KJ method, categorized science into "library science," "experimental science," and "field science."

 ・The KJ Method belongs to "Field Science," and its important characteristic lies in how hypotheses are abductively inferred and generated from experience and observation, rather than verified through experiments.

 ・In data processing, the fundamental concept of this method is to let the data speak for itself, rather than applying specific preconceptions or theories (the data tells us something).

 2) Analysis Process of the KJ Method (Ensuring Transparency)

 The following four stages are clearly described as the "specific procedures" requested by the reviewers.

 1. Labeling:

 ・Write data (facts + opinions) onto cards (labels).

 2. Grouping by Affinity

 ・Spread out the labels and repeatedly read them to sense the "affinity," grouping similar opinions together.

 ・Create a "Hyosatsu (Nameplate)" summarizing the contents of each group.

 ・Do not force classification; leave single items as they are.

 3. Spatial Arrangement :

 ・Arrange spatially based on the interrelationships between groups.

 ・This arrangement process is a critical task for logically explaining the observed phenomena.

 ・Lines and comments that explain the relationships are written, and the overall picture of the phenomena is illustrated.

 4. Writing (Synthesizing ):

 ・The text ) is composed to explain the diagram .

 ・It is the process of converting the spatial arrangement of the diagram into a logical narrative.

 3) Similarities with Grounded Theory (GT)

 1. Bottom-up theory construction

 Both extract concepts from various parts of the gathered data and structure them.

 2. Abstraction process through "comparison" and "integration"

 　Both compare data with each other, identify common properties, and categorize them.

 3. Deconstruction of the "existing context" through fragmentation

 　By first fragmenting the data into pieces, analysts overcome their preconceptions and existing contexts.　

 4. Orientation towards knowledge creation (theory building)

 It goes beyond merely organizing data (summarizing) and generates new meanings and theories from it.

 The reason for adopting the KJ method in this study was that it was necessary to first structure the complex situation in the field as it was, and the unique characteristics of this method contribute to the validity of the analysis.

 Regarding Strengthen Sampling Rationale and Refine Limitations Section(2,3)

 We understood the need to explain on the rationale for sampling, the limitations due to small sample size, and the study design that takes these limitations into account earlier in the paper. To address these points, we have rewritten part of the fourth paragraph in Chapter 3 as follows.

 “To understand current perceptions, and in light of Goto et al.’s (2025) focus on youth, we conducted interviews with recent graduates of School X who were in their first year of university at the time of the study.”

 ↓

 “In this study, interviews were conducted with recent graduates of School X who were first-year university students at the time of the survey. Goto et al. (2025), a prior study related to this research, conducted a detailed investigation focusing on young people. However, their survey questions emphasized the upward social mobility through marriage to men of the same or higher social status as the informants themselves, as well as cultivating the traditional ideal of the "good wife, wise mother." This is because it directly inherited the gender bias seen in prior researches such as Sato (2006) and Inoue (2018). On the other hand, the principal author of this study personally felt that the perspectives on gender held by the younger generation of Japanese differ from those of Sato(2006), Inoue(2018), and even Goto et al. (2025). Therefore, in this study, although some of the co-authors are the same as those in Goto et al. (2025), we aimed to minimize gender bias among researchers by assigning female co-authors as the first and second authors, in order to clarify the actual thoughts of contemporary female students.

 Interview participants were recruited through the personal network of the lead author. They shared the commonality of being graduates from the same Catholic integrated junior and senior high school and were first-year university students at the time of the survey, but no restrictions were placed based on family environment, parental beliefs, employment status, or other factors that could potentially influence their views on gender.”

 Additionally, the following sentence has been added to the seventh paragraph (in the new version) of Chapter 3.

 “It should be noted that the narrow sampling frame introduces limitations. The homogeneity of the group may restrict the diversity of perspectives, and the small sample size increases the risk of selection bias. To mitigate these risks, participants who graduated from the same high school but were enrolled in different majors at different universities were recruited. Nevertheless, the findings should be interpreted with these limitations in mind.”
